# Exploring the Nexus: A Systematic Review on the Interplay of the Methylenetetrahydrofolate Reductase (MTHFR) Gene C677T Genotype, Hyperhomocysteinemia, and Spontaneous Cervical/Vertebral Artery Dissection in Young Adults

**DOI:** 10.7759/cureus.60878

**Published:** 2024-05-22

**Authors:** Sajida Moti Wala, Esraa M AlEdani, Essa A Samuel, Khoula Ahmad, Naelijwa J Manongi, Ramkumar Rajapandian, Safeera Khan

**Affiliations:** 1 Internal Medicine, California Institute of Behavioral Neurosciences & Psychology, Fairfield, USA; 2 Dermatology, California Institute of Behavioral Neurosciences & Psychology, Fairfield, USA; 3 Physical Medicine and Rehabilitation, California Institute of Behavioral Neurosciences & Psychology, Fairfield, USA; 4 internal Medicine, California Institute of Behavioral Neurosciences & Psychology, Fairfield, USA; 5 Family Medicine, California Institute of Behavioral Neurosciences & Psychology, Fairfield, USA; 6 Trauma and Orthopaedics, California Institute of Behavioral Neurosciences & Psychology, Fairfield, USA

**Keywords:** hyperhomocystenemia, mthfr c677tmutation, spontaneous vertebral artery dissection, vitamin b 12 deficiency, young adult ischemic stroke

## Abstract

Ischemic strokes (IS) in young adults often evade early detection, resulting in delayed diagnosis until complications arise. Cervical/vertebral artery dissection, a significant contributor to these strokes, presents with symptoms such as migraine with aura, severe headache, and neck pain, commonly overlooked due to their nonspecific nature. This review investigates early indicators of artery dissections, emphasizing their importance in diagnosis and exploring the correlation between methylenetetrahydrofolate reductase (MTHFR) gene C677T genotype polymorphism, hyperhomocysteinemia (HHCY), and IS in young adults. This systematic review encompasses a thorough analysis of 11 papers, including two case-control studies, one retrospective observational study, one cohort study, three case reports, three narrative reviews, and one experimental study, involving a total of 4,840 patients aged 18-64 years*.* Findings reveal HHCY as a significant contributor to vascular damage and tissue ischemia leading to IS. The MTHFR gene C677T genotype polymorphism is closely associated with HHCY, often contributing to underdiagnosed strokes in young adults. Cervical/vertebral artery dissection may manifest as initial symptoms of neck pain or headache, remaining undiagnosed until imaging is conducted. Importantly, the review suggests that MTHFR gene polymorphism can be mitigated through simple supplementation with vitamin B12 and folates, serving as a valuable tool for primary prevention. Additionally, betaine, a methyl donor, was explored in severe MTHFR gene polymorphism cases resistant to conventional supplementation. In conclusion, recognizing the significance of early signs and symptoms, along with a high clinical suspicion, is crucial for preventing catastrophic outcomes, mortality, and morbidity associated with IS in young adults lacking traditional risk factors. The MTHFR gene C677T genotype polymorphism, a potential genetic cause, can be easily managed with simple measures but is often overlooked or underdiagnosed.

## Introduction and background

Constituting 71% of all strokes, ischemic stroke (IS) is a leading cause of both mortality and disability. Among young adults, aged 18-45 years or 18-50 years, IS accounts for 10 to 18% of all reported cases [1]. Hence, it is crucial to enhance our comprehension of the risks, causes, and treatment options for IS. Stroke is acknowledged a complex and polygenic disease, arising from a multitude of interactions between genes and both genetic and environmental elements [2,3]. Despite substantial progress in both prevention and treatment, stroke persists as a notable condition, standing as the foremost contributor to adult disability, the second principal cause of dementia, and the third leading factor in mortality rates within developed nations [3]. The incidence of stroke increases significantly with age, with relatively low rates in young adults. However, IS is a common reason for the admission of young patients to stroke units. In particular, the yearly occurrence of stroke escalates from 2.4 per 100,000 among individuals aged 20-24 years to 4.5 per 100,000 for those aged 30-34 years, and subsequently surges to 32.9 per 100,000 for individuals aged 45-49 years. Stroke prevalence is slightly higher in women aged 20-30 years and in men over 35 years of age [3]. Although traditional risk factors for stroke, such as diabetes and hypertension, alongside pathological conditions like small vessel disease, extensive intracranial and extracranial atherosclerosis and atrial fibrillation, wield significant influence in older patients, their occurrence is notably less frequent among young adults. The primary clinical challenge in managing stroke in young adults lies in identifying its cause, with a considerable proportion (35% to 42%) remaining undetermined [1,3]. In numerous instances, the underlying causes are related to genetics, and despite dedicated research endeavors, the genomic origins of IS continue to elude our understanding [2,3]. Genetic evaluation of individuals who have suffered a stroke can be one of the novel biological ways to understand the genetics behind these cerebrovascular events [3]. In this study, we plan to explore more on this very aspect of genetics behind IS.

Homocysteine (HCY) is an amino acid containing sulfhydryl, generated through the demethylation of methionine, an amino acid primarily found in animal protein. Plasma homocysteine levels can rise due to various factors, including age, deficiencies in vitamin B12, vitamin B6, and folate, renal dysfunction, and less commonly with smoking, arterial hypertension, hypercholesterolemia, and the consumption of coffee and alcohol. All these factors can interfere with methionine synthase activity causing pathologically elevated blood levels of homocysteine, known as hyperhomocysteinemia (HHCY), indicating a disruption in this biochemical process, leading to both biochemical and life-related consequences [3,4]. HHCY is reported to be independently associated with the risk of vascular events including stroke, by increasing the occurrences of atherosclerosis, thromboembolism, single vessel disease, or even arterial dissections [2,3,5]. One of the genes involved in HCY metabolism is the methylenetetrahydrofolate reductase (MTHFR) gene C677T polymorphism, which plays a critical role by reducing MTHFR activity and elevating HCY levels. The human MTHFR gene is situated on chromosome 1p36.3 [2]. The MTHFR serves as a crucial rate-limiting enzyme, facilitating the reduction of 5,10-methylenetetrahydrofolate to 5-methyltetrahydrofolate and deficiency of this enzyme, attributed to mutations in the MTHFR gene, can potentially predispose individuals to HHCY, disrupting the remethylation of HCY to methionine. Even a mild elevation causing HHCY poses a significant risk factor for atherosclerosis and vascular diseases, including stroke, ultimately contributing to the onset of cognitive impairment [6,7].

Amongst dissections, cervical artery dissections (CADs), including both carotid and vertebral arteries, may manifest as migraines or lead to strokes, some of which can be fatal. Arterial dissections involve a sudden tear in the wall of a major artery, resulting in the intramural collection of blood and these events constitute a group of severe disorders with catastrophic consequences, potentially resulting in major life-threatening outcomes. Thanvi et al. highlighted in their review article that the research conducted in the late 1970s played a pivotal role in identifying the clinical and radiological characteristics of dissection syndromes, thereby aiding in their antemortem diagnosis [8]. Advances in non-invasive imaging techniques have contributed to a rise in the identification and reporting of such dissections in individuals experiencing strokes. CADs are thought to contribute to up to 25% of strokes in individuals in young adults and middle-aged individuals (under the age of 50) [9,10]. The estimated prevalence of these dissections is currently at least 5 cases per 100,000 individuals, with over half of CAD patients eventually experiencing a stroke. Associations between CAD and metabolic factors include variations in MTHFR, a regulator of HCY levels. Changes in HCY levels are linked to thrombosis and atherosclerosis, particularly in cases of deficiencies in folate, vitamin B6, and vitamin B12. Notably, methionine adenosyltransferase 1A, a protein associated with aortic dissection, is also influenced by folate. It is also worth noticing that low plasma folate levels were found to increase the risk of CAD in a cohort of 39 patients, adding an intriguing dimension to these associations [9].

In this systematic review, our objective is to establish a connection between the MTHFR gene C677T polymorphism, which induces HHCY and consequently leads to spontaneous cervical or vertebral artery dissections in otherwise healthy young adults without known risk factors. Additionally, we seek to investigate the significance of recognizing early signs and symptoms of arterial dissections in young individuals. These individuals often present with signs and symptoms of IS, which are frequently overlooked in diagnosis. Furthermore, we also aim to delve into the role of vitamin B12 and folate in the physiology of individuals with MTHFR enzyme deficiency, exploring how early supplementation or treatment can significantly decrease the incidence of stroke in the at-risk population.

## Review

Methodology

We used Preferred Reporting Items for Systemic Review and Meta-Analysis (PRISMA) 2020 guidelines for conducting this systematic review [11].

Search Sources and Strategy

We searched PubMed/Medline, PubMed Medical Subject Heading (PubMed MeSH), PubMed Central (PMC), Cochrane Library, Google Scholar, and Science Direct to search for the relevant literature. We used various combinations of MTHFR (c677t genotype) hyperhomocysteinemia, spontaneous vertebral artery dissection, and stroke/transient ischemic attack (TIA) to conduct a comprehensive search across all databases. In PubMed, we used the following strategy to search relevant literature in PubMed’s MeSH database, along with the above keywords.((“Hyperhomocysteinemia/classification"[Mesh] OR "Hyperhomocysteinemia/complications"[Mesh] OR"Hyperhomocysteinemia/genetics"[Mesh] )) AND ("Methylenetetrahydrofolate Reductase (NADPH2)"[Mesh] AND ( "Methylenetetrahydrofolate Reductase (NADPH2)/biosynthesis"[Mesh] OR "Methylenetetrahydrofolate Reductase (NADPH2)/deficiency"[Mesh] OR "Methylenetetrahydrofolate Reductase (NADPH2)/genetics"[Mesh] )) AND (( "Vertebral Artery Dissection/complications"[Mesh] OR "Vertebral Artery Dissection/diagnosis"[Mesh] OR "Vertebral Artery Dissection/etiology"[Mesh] )). Table 1 illustrates the databases employed and the respective quantities of identified papers for each database.

**Table 1:** Keywords used for search strategies and the number of papers identified. MeSH: Medical Subject Heading, PMC: PubMed Central

SEARCH STRATEGY	DATABASE USED	NO OF THE PAPERS IDENTIFIED
(( "Hyperhomocysteinemia/classification"[Mesh] OR "Hyperhomocysteinemia/complications"[Mesh] OR "Hyperhomocysteinemia/genetics"[Mesh] )) AND ("Methylenetetrahydrofolate Reductase (NADPH2)"[Mesh] AND ( "Methylenetetrahydrofolate Reductase (NADPH2)/biosynthesis"[Mesh] OR "Methylenetetrahydrofolate Reductase (NADPH2)/deficiency"[Mesh] OR "Methylenetetrahydrofolate Reductase (NADPH2)/genetics"[Mesh] ))	PubMed MeSH	446
("Methylenetetrahydrofolate Reductase (NADPH2)"[Mesh] AND ( "Methylenetetrahydrofolate Reductase (NADPH2)/biosynthesis"[Mesh] OR "Methylenetetrahydrofolate Reductase (NADPH2)/deficiency"[Mesh] OR "Methylenetetrahydrofolate Reductase (NADPH2)/genetics"[Mesh] )) AND (( "Vertebral Artery Dissection/complications"[Mesh] OR "Vertebral Artery Dissection/diagnosis"[Mesh] OR "Vertebral Artery Dissection/etiology"[Mesh] ))	PubMed MeSH	4
( "Hyperhomocysteinemia/classification"[Mesh] OR "Hyperhomocysteinemia/complications"[Mesh] OR "Hyperhomocysteinemia/genetics"[Mesh] )) AND (( "Vertebral Artery Dissection/complications"[Mesh] OR "Vertebral Artery Dissection/diagnosis"[Mesh] OR "Vertebral Artery Dissection/etiology"[Mesh] ))	PubMed MeSH	8
methylenetetrahydrofolate reductase deficiency AND hyperhomocysteinemia	PubMed	169
hyperhomocysteinemia AND stroke AND methylenetetrahydrofolate reductase deficiency		
hyperhomocysteinemia AND vertebral artery dissection		
("hyperhomocysteinemia"[MeSH Terms] OR "hyperhomocysteinemia"[All Fields]) AND ("Methylenetetrahydrofolate reductase deficiency"[All Fields] OR "methylenetetrahydrofolate reductase deficiency"[All Fields])	PMC / Medline	340
("hyperhomocysteinemia"[MeSH Terms] OR "hyperhomocysteinemia"[All Fields]) AND ("vertebral artery dissection"[MeSH Terms] OR ("vertebral"[All Fields] AND "artery"[All Fields] AND "dissection"[All Fields]) OR "vertebral artery dissection"[All		
MTHFR AND homocysteine	Cochrane library	108
Methylenetetrahydrofolate AND artery dissection	Google Scholar	386
MTHFR c677t polymorphism AND hyperhomocysteinemia AND ischemic attack	Science Direct	184
MTHFR c677t polymorphism AND hyperhomocysteinemia AND artery dissection		
Total papers identified		1645
Total papers after removing duplicates		1114

Inclusion and Exclusion Criteria

We included papers from the last 10 years, papers written and published in the English language, and human participants for study purposes. We excluded articles if the full-text literature could not be retrieved. Those involving comorbid conditions and a family history of genetic disease/ chronic illness/ pregnant or cancer patients/chiropractic manipulation were also not included. Articles having grey literature were also excluded from the study.

Selection Process

We first used Endnote to transfer our shortlisted articles and removed any duplicate papers. Henceforth, each article was screened through titles, abstracts, and conclusions and independently assessed by first and second authors. The articles shortlisted were then assessed using our inclusion/exclusion criteria and any conflicts were then discussed amongst the co-authors by evaluating the full text. The selected articles were then evaluated to be included in the study.

Quality Assessment of the Studies

The selected articles underwent quality assessment through appropriate quality appraisal tools, with all co-authors participating in the evaluation process. The quality of observational studies was evaluated using the Newcastle-Ottawa tool, while narrative reviews were assessed using the Scale for the Assessment of Narrative Review (SANRA), and case reports were appraised using the Joanna Briggs Institute (JBI) tool. Only studies meeting the criteria of the quality appraisal were included in the systematic review.

Data Collection Process

After the articles were chosen for the systematic review and extracted, the primary outcomes, along with crucial details, underwent evaluation. The initial data extraction was independently conducted by the first author, with all authors contributing equally to finalize the gathered data and analyze the outcomes based on the data extraction questionnaires.

Results

Study Identification and Selection

**Figure 1:**
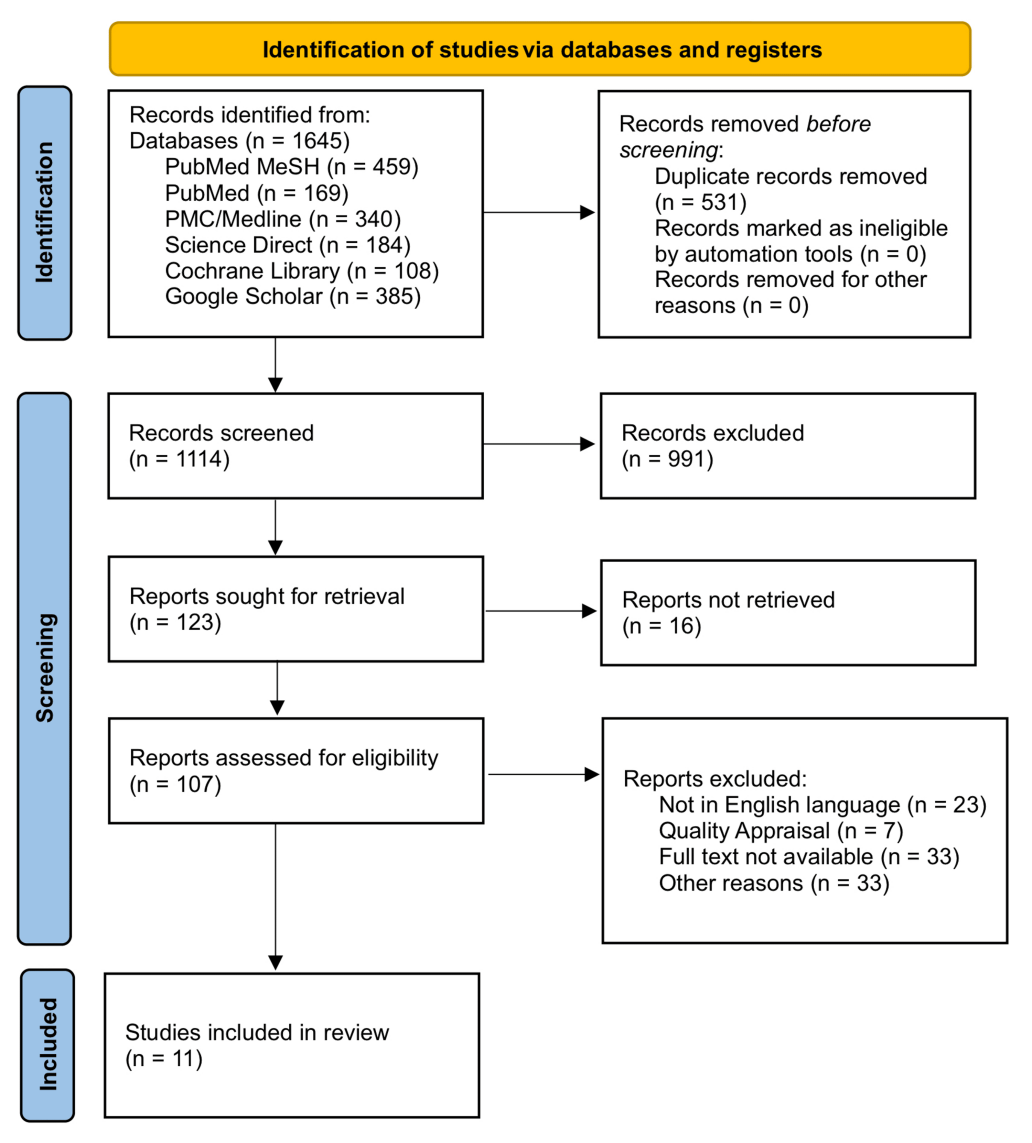
PRISMA 2020 flowchart depicting the process for article selection. PRISMA: Preferred Reporting Items for Systematic review and Meta-Analysis, MeSH: Medical Subject Heading, PMC: PubMed Central

A total of 1645 relevant articles were detected, using all databases. In total, 531 duplicate articles were removed. We shortlisted a total of 107 articles by going through titles and abstracts. From the shortlisted articles, the ones which did not meet our eligibility criteria and quality or for which the full text could not be retrieved were removed, and 11 articles were finalized for review. The study selection process is depicted in Figure 1 of the PRISMA flowchart. 

The eligibility of the articles was evaluated using various quality appraisal tools as shown below. Table 2 shows quality appraisal using the Newcastle Ottawa tool. Table 3 shows quality appraisal using the JBI tool. Table 4 shows quality appraisal using the SANRA checklist. 

**Table 2:** Quality appraisal by using Newcastle Ottawa tool

Studies	Selection	Comparability	Outcome
Jazbec et al., 2022 [1]	****	*	**
Kumar et al., 2016 [2]	****	*	**
Aifen li et al., 2017 [5]	****	*	**
Ahmed et al., 2019 [12]	****	*	***

**Table 3:** Quality appraisal using the Joanna Briggs Institute (JBI) tool.

Study characteristics	Vieira et al., 2020 [6]	Elkady et al., 2020 [13]	Chen et al., 2018 [14]
Were the patient's demographic characteristics clearly described?	Yes	Yes	Yes
Was the patient’s history clearly described and presented as a timeline?	Yes	Yes	Yes
Was the current clinical condition of the patient on presentation clearly described?	Yes	Yes	Yes
Were diagnostic tests or assessment methods and the results clearly described?	Yes	Yes	Yes
Was the intervention(s) or treatment procedure(s) clearly described?	Yes	Yes	Yes
Was the post-intervention clinical condition clearly described?	Yes	Yes	Yes
Were adverse events (harms) or unanticipated events identified and described?	unclear	unclear	Yes
Does the case report provide takeaway lessons?	Yes	Yes	Yes

**Table 4:** Quality appraisal using the Scale for the Assessment of Narrative Review (SANRA) checklist.

Study characteristics	Eva et al., 2015 [3]	Cordaro et al., 2021 [4]	Bax et al., 2022 [9]
Justification of the article’s importance for the readership.	2	2	2
Statement of concrete aims or formulations of questions.	2	1	1
Description of the literature search.	0	0	0
Referencing.	2	2	2
Scientific reasoning.	2	2	2
Appropriate presentation of data (e.g. Absolute risk vs relative risk; effect sizes with confidence interval)	Not applicable	Not applicable	Not applicable

Outcomes Measured

The key outcomes extracted from the finalized research papers focused on the role of MTHFR gene mutation causing HHCY. The secondary outcomes assessed were vertebral/carotid artery dissections in otherwise healthy and young adults, causing IS or TIA without any known underlying risk factor. Some studies explored the effect of vitamin B12, and B6 supplementation in MTHFR gene mutation, in reducing HHCY and consequent episodes of stroke in young individuals, and as a potential treatment option for the same.

Study Characteristics

We reviewed 11 research papers comprising a total of 4,840 patients. From these finalised studies, we identified two case-control studies [2,5], three case reports [6,13,14], three narrative reviews [3,4,9], one retrospective observational study [1], one cohort study [12], and one experimental study [15]. All the studies include young patients presenting to the hospital with either TIA or stroke, evaluated to identify HHCY and further MTHFR gene C677T polymorphism, or patients with cervical/vertebral artery dissections, presenting with cervicogenic headache, which were often initially misdiagnosed as migraine with aura, until later returning with more serious outcomes. Few studies explored the genetics behind IS, and others examined the association between MTHFR gene C677T polymorphism and HHCY in the literature. Almost all studies concluded that vitamin B12, vitamin B6, and folic acid supplementation can drastically improve outcomes in patients with MTHFR gene C677T polymorphism. Table 5 depicts a summary and characteristics of all studies included.

**Table 5:** Summary of included studies. CAD: cervical artery dissection; TIA: transient ischemic attack; IS: ischemic stroke; MTHFR: methylenetetrahydrofolate reductase; SVD: small vessel disease; GWAS: Genome-wide Association Study; Met-B12: methyle-B12; VAD: vetebral artery dissection; HHCY: hyperhomocysteinemia; HCY: homocysteine; FA: folic acid; 5-Me-THF: 5-methyltetrahydrofolate

Authors and year of publication	Study Type	Purpose Of The Study	No. Of Participants	Results	Conclusions
Jazbec et al. 2022 [1]	Retrospective Observational study	To establish a correlation between CAD leading to IS in young population	196	The study included 196 patients with IS, without any known traditional risk factors for stroke, and found dissection to be present in 16 cases(8.2%)	IS is a common presentation in young adults with artery dissection and should not be missed in the absence of traditional risk factors
Kumar et al. 2016 [2]	Case-control study	To determine the association between MTHFR C677T (rs1801133) gene polymorphism and risk of IS in the North Indian population	250	Study showed an independent association between MTHFR polymorphism and IS in North Indian population. The Mean age of cases and controls were selected as 52.83 ± 12.59 and 50.97± 12.70 years. MTHFR C677T gene polymorphism was independently linked to the risk of SVD after adjustment for potential confounding factors	The findings in the conducted study suggest an independent association between MTHFR gene polymorphism and a risk factor in IS, especially in SVD subtypes in the North Indian population
Eva et al. 2015 [3]	Narrative Review	To study the genetics of IS in young adults	0	The identiﬁcation of a genetic cause is important to determine the etiology of IS in young adults to give suitable counseling and to initiate the correct therapy, when available	The introduction of GWAS technology, as seen in various complex pathological conditions, has markedly enhanced our understanding of many of the genetic bases of IS
Cordaro et al. 2021 [4]	Narrative Review	To know the possible role of HHCY in neurodegenerative disorders	0	Metabolism/catabolism of methionine and HCY relies on intricate biochemical pathways involving multiple enzymes and yielding diverse molecules crucial for cell survival. Numerous studies have reported that the supplementation of B- complex vitamins, folate, and other compounds involved in the metabolic pathway of methionine has been beneficial in mitigating the severity of HHCY, thereby aiding in various pathological conditions	Currently, HHCY is not only recognized as a diagnostic marker for various pathologies, but its potential as a therapeutic target is also under consideration and while its role in neurodegenerative disease is evident from a large no.of studies, it is still far from being considered a biomarker of the same
Aifen li et al. 2017 [5]	Case control study	To study the influence of serum total homocysteine levels, MTHFR C677T polymorphisms, and their interconnection in the risk of IS within a central Chinese Han population	300	Plasma homocysteine was significantly higher in patient with IS with MTHFR polymorphism TT>CC and CT genotype	A potential synergistic impact of the MTHFR C677T polymorphism on variations in homocysteine levels is suggested to contribute to an elevated risk of IS in the Chinese population
Vieira et al. 2020 [6]	Case report	To study a case of adult-onset MTHFR enzyme deficiency leading to IS	1	A young patient presenting as a case of IS without any known risk factors was evaluated and found to have HHCY due to MTHFR gene polymorphism	Inborn errors of metabolism may have a late-­onset presentation and severe HHCY may have neurological and psychiatric manifestations. Patient prognosis depends on early diagnosis and specific treatment
Bax et al. 2022 [9]	Narrative review	To know the risk factors leading to an arterial dissection, common symptoms and findings	0	Despite varying risk factors, the basic pathology behind a dissection remains the same	Cellular activation is a significantly underexplored mechanism in arterial dissections, and it could potentially serve as a crucial therapeutic target
Ahmed et al. 2019 [12]	Cohort study	Vitamin B 12 deficiency and HHCY in outpatients with stroke or TIA	4055	HHCY and Met-B12 deficiency was found in a majority of patients presenting with stroke/TIA	Vitamin B12 can help significantly to lower the serum HHCY which is one of the major risk factors for strokes and cognitive impairment. Supplementation of B12 can help lower the incidence of stroke in the high-risk population
Elkady et al. 2020 [13]	Case Report	To assess the atypical presentation of VAD in young adults	2	Spontaneous VAD may begin with vague symptoms which are easily misdiagnosed in young adults	High suspicion for a spontaneous VAD should be kept in young patients presenting with cervicogenic headaches or those with first episodes of headache with visual disturbances mimicking a migraine with aura
Chen et al. 2018 [14]	Case Report	To study the atypical presentation of postural headache in a young adult with spontaneous VAD	1	Differential diagnosis for postural headache should always be evaluated in young patients with no obvious risk for intracranial hypotension	Orthostatic headache may mimic VAD as initial clinical presentation, carotid echo can help establish an early diagnosis, and timely intervention can help in preventing a massive hemorrhage
Vidmar Golja et al. 2020 [15]	Experimental study	To assess the impact of folate deprivation and supplementation on the cell growth and proliferation of human lymphoblastoid cells, as well as to evaluate the effectiveness of FA or 5-Me-THF supplementation in enhancing the intracellular concentration of biologically active folate in genetically diverse cells	35	A deficiency in folate leads to cell cycle arrest in the S-phase. Supplementation with folate enhances cellular metabolic activity. 5-Me-THF exhibits greater efficacy than FA in boosting the cellular metabolism in instances of low MTHFR activity. MTHFR deficiency impacts intracellular concentration of 5-Me-THF	Cells cultured in folate-deprived media displayed diminished metabolic activity and slower proliferation compared to controls cultured in media supplemented with 50 nM folate. Moreover, 5-Me-THF can circumvent folate insufficiency induced by MTHFR deficiency

Discussion

Diagnosing stroke in young adults is often challenging due to the presence of other conditions that can mimic its signs and symptoms, with migraine being one of the most common culprits. The absence of traditional risk factors in young adults makes the diagnosis even more difficult, increasing the risk of missed diagnoses and potentially debilitating consequences. One of the significant risk factors for stroke in young adults is HHCY leading to CAD or vertebral artery dissection (VAD) and overlooking this factor can lead to catastrophic outcomes. Therefore, it is crucial to maintain a high level of clinical suspicion when evaluating such cases. Research has consistently shown that early diagnosis and management are key factors in preventing morbidity and mortality.

Pathophysiology of Hyperhomocysteinemia

**Figure 2:**
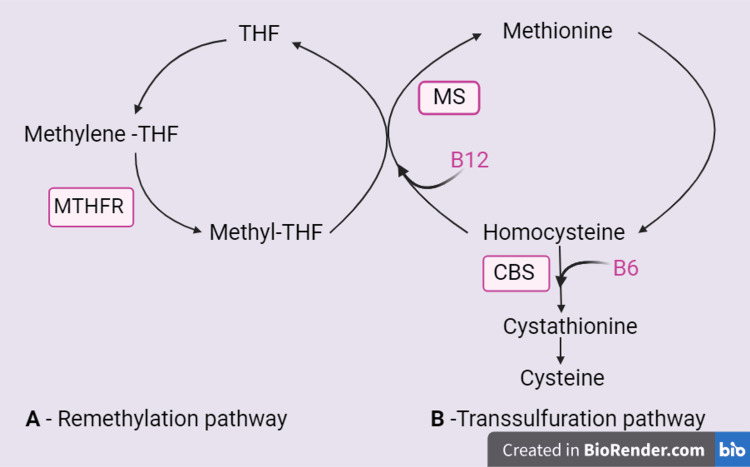
Metabolism of homocysteine. A- Remethylation pathway. B- Transsulfuration pathway. THF: tetrahydrofolate, MTHFR: methylene-tetrahydrofolate reductase, MS: methionine synthase, CBS: cystathionine beta synthase, B12: vitamin B12, B6: vitamin B6. Figure created by the corresponding author (SMW).

HCY is an amino acid containing a sulfhydryl group, produced through the demethylation process of methionine, an amino acid predominantly found in animal protein [4]. The majority of HCY in the body originates from methionine found in both plant and animal proteins. About 3% of HCY exists in the body in a free state, binding with other molecules or in disulfide forms. The body employs a pathway dependent on folic acid and vitamins B6/B12 to recycle methionine by converting HCY back. HHCY often results from deficiencies in folate or cobalamin (vitamin B12) or mutations in crucial enzymes such as MTHFR and cystathionine beta-synthase (CBS). The severity of HHCY is categorized based on plasma HCY levels, considered severe if >100 micro mol/L, intermediate at 31-100 micro mol/L, and moderate at 16-30 micromol/L. Variations in metabolism can be influenced by specific dietary and lifestyle factors, including increased coffee consumption, cigarette smoking, and alcohol abuse, which can disrupt methionine synthase activity. HHCY results in oxidative stress, causing cellular damage (endothelial and vascular smooth muscle cells) primarily because it leads to a decline in mitochondrial respiration, characterized by decreased activities of electron transport chain (ETC) complexes and a reduction in ATP production [4,5,12]. The effective removal of excess HCY from intracellular environments to the systemic circulation is facilitated by liver and kidney excretion, involving enzymes such as CBS and betaine HCY methyltransferase (BHMT), which convert HCY into non-toxic metabolites. Also, recent studies have implicated HHCY in several medical conditions such as Alzheimer's, Parkinson's, vascular dementia, stroke, epilepsy, and more, all of which are currently undergoing extensive global research for early preventive measures [4,16]. Deficiencies in crucial elements such as vitamin B12, folate, or vitamin B6, along with mutations in enzymes like MTHFR or CBS, can cumulatively lead to disrupted HCY metabolism. This is because the de novo synthesis of methionine methyl groups necessitates both folate and vitamin B12 co-factors, while the production of cystathionine relies on pyridoxal 50-phosphate (vitamin B6) [4]. A crucial opportunity for preventing stroke and dementia lies in addressing metabolic B12 deficiency [12]. This often goes unnoticed because many physicians are not aware that a serum total B12 within the 'normal' range (~180-670 pmol/L) does not necessarily indicate sufficient functional vitamin B12 levels. To evaluate B12 function accurately, it's essential to measure holotranscobalamin or one of the metabolites that show an increase in B12 deficiency: methylmalonic acid (MMA) or total homocysteine (tHCY). While MMA is more specific, HCY can serve as a substitute in individuals with sufficient folate levels (which, in countries with folate fortification, is applicable to nearly all patients). The threshold for serum B12, at which both MMA and tHCY rise, is 400 pmol/L. Consequently, within the normal range of serum B12, numerous patients may have metabolic B12 deficiency [12]. Figure 2 shows the metabolism of homocysteine via remethylation and transsulfuration pathways.

Role of the MTHFR Gene in HHCY

Following its biochemical characterization in 1991 and genetic identification in 1995, the 677C>T allele (T allele) of the MTHFR gene has garnered growing attention from researchers worldwide [17]. To understand the importance of these enzymes we need to improve our understanding of folate metabolism. Dietary folates primarily exist in the form of polyglutamates and must undergo deconjugation to monoglutamates before absorption. The enzyme glutamate carboxypeptidase II (GCPII) accomplishes this in the small intestine. Monoglutamate forms of folate, such as folic acid (FA), 5-Me-THF, and 5-formyl-tetrahydrofolate, are actively transported across enterocytes via two transporters: the reduced folate carrier (RFC or SLC19A1) and the proton-coupled folate transporter (PCFT). Converting monoglutamates back to polyglutamates ensures the retention of folate within the cell, preventing loss through efflux [15].

To enter the folate cycle, folate polyglutamates must first be converted to dihydrofolate (DHF). Subsequently, they are transformed into the active form, 5-Me-THF, the primary folate metabolite in the blood. This conversion is facilitated by the enzyme 5,10-MTHFR, a crucial player in folate and HCY metabolism. It catalyzes the reduction of 5,10-MTHFR to 5-Me-THF, providing the methyl group necessary for the remethylation of HCY to methionine, a process during which tetrahydrofolate (THF) is generated [15]. This product plays a crucial role as a methyl group donor in the remethylation process of HCY to methionine. Methionine, in turn, is essential for the synthesis of S-adenosyl methionine (SAM) through the methionine cycle. SAM serves as a primary provider of methyl groups for numerous biological methylation reactions, essentially contributing to the metabolism of RNA, DNA, lipids, proteins and hormones [2,18]. In conditions with MTHFR gene polymorphism, mostly a single base pair transition (677C/T) in the MTHFR gene results in the change of the amino acid residue from alanine to valine. This alteration influences the enzyme's thermo-lability, causing a 30% decrease in enzyme activity [2]. Consequently, this leads to elevated levels of HCY and hypomethylation in the homozygous state, contributing to HHCY, which is known to be independently associated with an increased risk of vascular events, including stroke [2,3]. 

Apart from dietary intake, genetic predisposition also plays a role in modulating plasma levels of folates. Common polymorphisms in genes encoding enzymes of the folate cycle are prevalent, with many having a minor allele frequency (MAF) of over 25% in the general population. Among these, the most crucial ones are polymorphisms of the 5,10-MTHFR gene. Notably, two functional polymorphisms, 677C>T (rs1801133) and 1298A>C (rs1801131) significantly decrease the activity of MTHFR and are linked to decreased plasma levels of biologically active folate [15]. Based on ethnicity, striking differences were seen in the frequencies of MTHFR polymorphisms, while African-Americans seem to exhibit a level of protection against MTHFR deficiency, Hispanics and Caucasians seem to be at an increased risk, with higher frequencies of 677C>T and 1298A>C, respectively, as reported in a cohort study of 1405 patients by Graydon et al. [7]. In another comparable study examining Asian and Caucasian populations, the strength of association was observed to be greater in the Asian population than in the Caucasian population [2].

Mechanism of Spontaneous Arterial Dissection, Its Correlation With HHCY and Its Outcome of IS

Arterial dissection refers to a structural failure in the arterial wall, leading to an intramural bleed that forms an intramural hematoma (IMH), causing a dissection of the vessel wall. As the IMH enlarges, it induces the ipsilateral vessel wall to protrude into the vessel lumen towards the contralateral wall. In vessels with smaller diameters, this bulging action obstructs blood flow, preventing tissue perfusion and resulting in ischemia and/or infarction. If the dissection is coupled by an intimal tear, the true lumen may become obstructed because the IMH can form a thrombus that extends into and blocks it. Alternatively, it may result in emboli that occlude distal arterial branches, leading to the formation of micro-infarcts. Dissections primarily occur in medium and large arteries, with their frequency decreasing as arterial size diminishes. From a clinical perspective, dissections are frequently categorized based on their anatomical location, determining the medical specialty most relevant to the dissection. For instance, cervical dissections are the focus of neurologists, while aortic dissection falls under the purview of cardiologists. The origin of dissections is believed to be in two of the three arterial layers: the innermost layer, consisting of a monolayer of endothelial cells (EC), known as the tunica intima; while the thick muscular middle layer, called the tunica media, consists of concentric layers of vascular smooth muscle cells (VSMCs) and extracellular matrix (ECM), forming the lamellar unit. It's noteworthy that the outermost layer, known as the ECM covering (tunica adventitia), doesn't contribute to the start of dissections [9].

**Figure 3:**
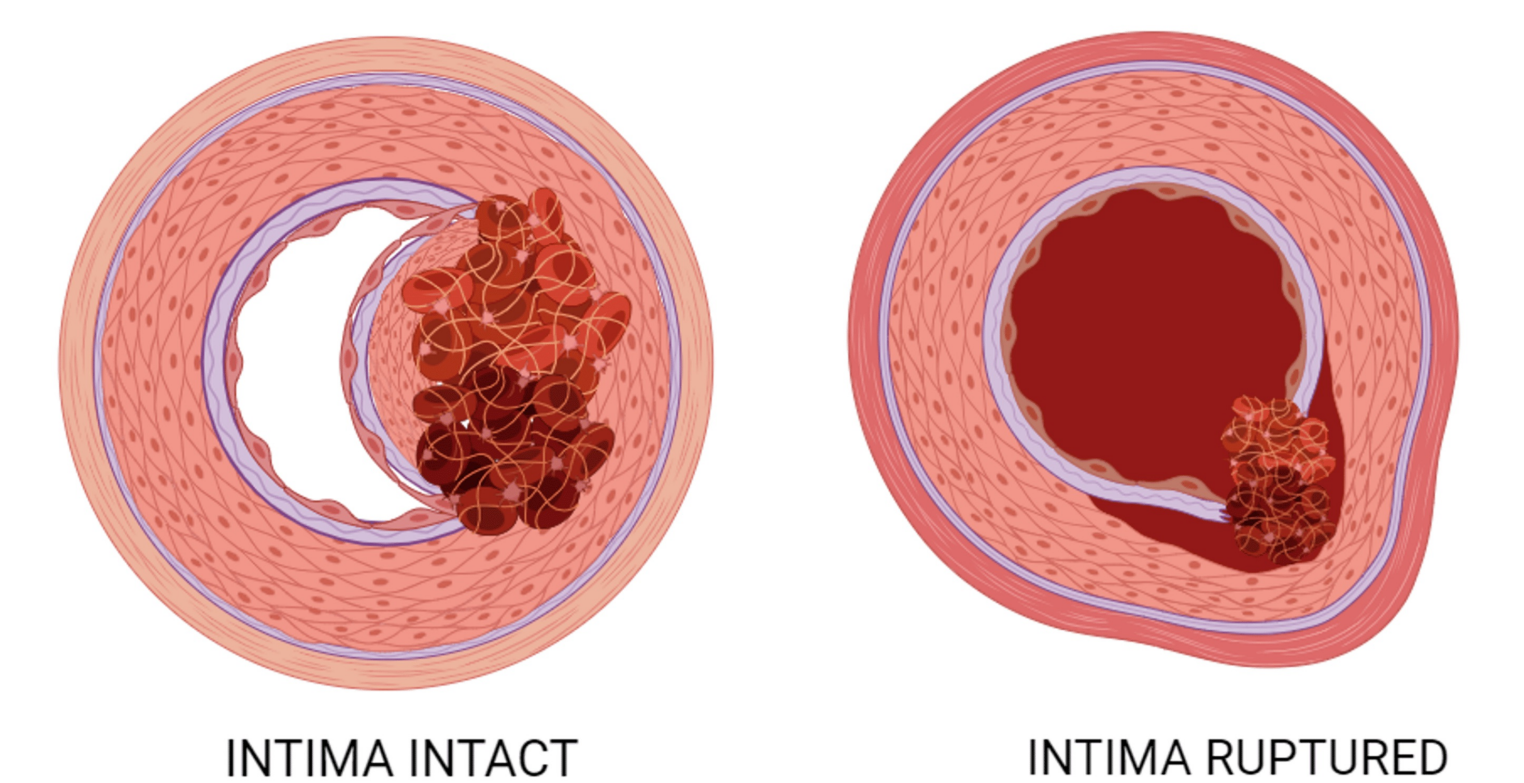
Cross-section of an artery depicting both pathologies of arterial dissection: 1. Bleeding within the tunica media resulting in narrowing of the lumen of the artery, can also cause compression of the neighbouring vessels, thereby preventing tissue perfusion. 2. Intimal flap rupture leading to formation of intramural hematoma (IMH). Figure created with BioRender.com by the corresponding author (SMW).

Dissections have not been identified in resistance vessels. The branching pattern of arteries adds complexity to the vascular structure, creating unique arterial microenvironments. For instance, the nonlinear morphology of vessels, characterized by bifurcations or divergent intersections, exposes various arterial areas to experiencing different blood flow forces, specifically shear stress. The ability of arteries to uphold the suitable levels of elasticity and flexibility for proper flow at both tissue and cellular levels depends on factors such as ECM composition, physical properties, structural elements, and metabolic proteins. Environmental factors such as age, sex and lifestyle influence the expression of these proteins. Arterial dissections occur when the structural integrity of arteries is compromised. With the exception of aortic dissections, the development of an IMH hinders perfusion through the artery [9]. Dissection can be classified into two groups with two distinct pathologies: 1. Bleeding within the tunica media forming IMH, resulting in narrowing of the lumen of the artery, can also cause compression of the neighboring vessels, thereby preventing tissue perfusion, and 2. Intimal flap rupture leading to the formation of IMH. Figure 3 depicts the two pathologies of arterial dissection.

Any of the above pathologies can lead to spontaneous arterial dissections, which may manifest either linked to connective tissue disorders or occurring independently, impacting seemingly healthy individuals. A widely acknowledged theory proposes a two-hit model for spontaneous arterial dissections, involving a genetic predisposition as the first hit and an environmental trigger as the second hit to precipitate the event. These dissections may manifest in various anatomical locations, including the cervical arteries and aorta, and there can be multiple dissections in a single area, indicating the existence of mechanistic subtypes of arterial dissection. For our study, we specifically focussed on CADs, which involve dissections of the vertebral and carotid arteries and can manifest as migraines or lead to potentially fatal strokes. CADs are considered responsible for as much as 25% of strokes in young and middle-aged adults (under 50). The estimated prevalence of these dissections is at least five cases per 100,000 individuals, with over half of CAD patients eventually developing a stroke [9]. CADs typically manifest with an IMH, which may or may not be linked with an intimal flap tear, occasionally accompanied by the presence of pseudoaneurysms. In cases involving an intimal tear, the endothelium is frequently characterized as irregular. Notably, a study examining connective tissue disorders in 65 patients with CAD revealed that 55% exhibited microscopic abnormalities in collagen, akin to those observed in Ehlers-Danlos syndrome [9]. Strikingly, only 5% of these patients displayed clinical signs of skin, joint, or skeletal abnormalities. These results distinctly suggest that defects specific to the vascular ECM play a crucial role in the etiology of the disease. In a study focused on CAD patients, sexual dimorphism was apparent, with 57% of the participants being male. Remarkably, the remaining 43%, consisting of females, were notably younger, averaging 42.5 years, compared to 47.5 years for male patients. The extent to which referral bias contributed to this divergence in patient populations remains unclear, but hypertension and migraine are recognized risk factors for CAD. Additionally, acute infection has been identified as an elevated risk factor for CAD, with nearly one-third (31.9%) of cases reporting acute infection within one month prior to CAD, compared to 13.5% in the control group [8,9]. Notably, in one of Guillon et al.'s case-control studies, it was observed that acute infections are more commonly associated with multiple dissections than with single artery dissection [19].

Genetic risk factors for CAD are relatively understudied. Mutations in the genes encoding intercellular adhesion molecule 1 (ICAM1) and cytoskeletal alpha-1-syntrophin (SNTA1) have been linked to CAD. Additionally, associations between CAD and metabolism, involve variants in the MTHFR enzyme. As discussed earlier, alterations in HCY levels are linked to thrombosis and atherosclerosis, conditions that arise with deficiencies in vitamin B12, vitamin B6 and folate. Ahmed et al.'s retrospective study on 4055 patients also provides evidence of a substantial correlation between IS and vitamin B12 deficiency, leading to elevated total HCY levels [14]. Decreased plasma folate levels were associated with an elevated risk of CAD in a cohort of 39 patients, as documented by Bax et al. in their study [9]. The retrospective study conducted by Park et al., which involved 146 patients diagnosed with arterial or venous thromboembolic disease, provides additional evidence of a thrombotic tendency associated with HHCY resulting from the MTHFR gene C677T polymorphism [20].

As evident by several studies, HHCY levels might result in changes in cerebral blood flow, potentially leading to thrombosis and alterations in cerebral oxygen transfer, ultimately contributing to the occurrence of migraine aura events. Possible factors include vasodilation or transient thrombosis in homocysteine-induced cerebral blood vessels. Moreover, HHCY contributes to a hypercoagulable state, evidenced by increased von Willebrand factor or prothrombin activation. The association between HCY and hypercoagulation may provide insight into the elevated risk of stroke and cardiovascular events in these individuals. Additionally, HCY may contribute to the onset of migraines by generating superoxide anions, leading to oxidative damage to the vascular endothelium and hence this forms the primary presenting complaint of most individuals [4,21]. Elkady et al., in their case studies, concluded that presentations of CAD and VAD are frequently subject to misdiagnosis, and diagnosis is often delayed until ischemic events occur [13]. They observed that genetic connective tissue conditions, migraine with aura, hypertension, HHCY, and a recent history of infections were reported more frequently in patients with VAD compared to healthy controls. The authors also suggested that spontaneous VAD should be strongly suspected in patients experiencing the initial episode of severe cervicogenic headache, particularly those with a history of minor trauma. Additionally, it should be considered in patients presenting with the first episode of headache accompanied by visual disturbances resembling migraine with aura [8,9].

Similarly, in another case report by Chen et al., similar findings were observed in a 37-year-old Taiwanese patient [14]. The conclusion drawn was that orthostatic headache has the potential to mimic VAD in the initial clinical presentation, emphasizing the importance of maintaining a high suspicion in patients presenting with such symptoms. Orthostatic headache is traditionally linked to intracranial hypotension caused by cerebrospinal fluid (CSF) leak. Despite its incidence being approximately 1% of all emergency room headaches, it remains an important consideration as a potential differential diagnosis.

In a retrospective study conducted by Jazbec et al., involving 196 individuals with IS, dissection of cervical arteries was identified in 16 cases (8.2%) [1]. The study concluded that CAD is the most common independent cause, representing approximately 20% of all cases of IS in young adults. The participants in their study were aged between 18 and 49 years and lacked traditional risk factors for IS. Another study from Colombia reported CAD as one of the main causes of IS in young adults, with 23.6% of IS patients being diagnosed with CAD. In an American study, 23.7% of cases involved CAD among patients with IS, and a Helsinki study reported 15%. Another study from the United States indicated that dissection emerged as the predominant cause within the 'other determined cause' category for young adults, contributing to 13% of ischemic infarctions in patients aged 18 to 45 years and up to 20% in those under the age of 25 [1].

In another case-control study conducted by Kumar et al. in North India involving 250 patients, it was established that HHCY is associated with the subtype of stroke known as small vessel disease (SVD) and large vessel disease (LVD) [2]. Similar reports were found in a case-control study conducted on 300 patients by Li et al. in China [5]. Both the studies utilized the polymerase chain reaction-restriction fragment length polymorphism (PCR-RFLP) technique to detect genotypes of the MTHFR gene C677T, demonstrating a significant association between the C677T polymorphism of the MTHFR gene and an elevated risk of IS in the North Indian population and Central Chinese Han population. Chen et al., in their retrospective cohort study, also concluded similar findings, with a significantly higher association between HHCY and SVD in patients compared to the control group [22]. 

These findings were consistent with a meta-analysis comprising 38 studies involving 6,310 cases and 8,297 controls, which also demonstrated a notable correlation between MTHFR gene C677T polymorphism and the risk of IS. Another meta-analysis, encompassing 68 case-control studies with 7,990 cases and 6,941 controls from the Chinese population, reaffirmed the substantial association between MTHFR gene C677T polymorphism and the risk of cerebrovascular diseases (OR 1.81, 95% CI 1.6-1.9: TT vs. CC) [2].

In one of their case reports, Vieira et al. describe a 23-year-old male who presented with insidious dysarthria, gait impairment, and numbness in both legs [6]. Despite symptomatic treatment with B12 and folate supplementation, there was no improvement. Further investigation revealed an MTHFR gene mutation in this individual. The addition of betaine, a specific methyl group donor, to the treatment regimen resulted in improvement. This highlights betaine as the only treatment option yet explored for severe HHCY caused by gene mutations, particularly MTHFR mutations. Betaine, as a methyl donor, circumvents the need for the MTHFR enzyme [6,23]. By doing so, it reduces the risk of HHCY, thereby mitigating the associated consequences such as arterial dissections and the dreaded outcome of stroke. Similarly, Biesalski et al. demonstrated significant improvement in their 30-year-old patient, who had late-onset severe MTHFR deficiency with complete loss of vision, severe spastic tetraparesis, cognitive impairment, pronounced bradyphrenia, and therapy-refractory seizures, using high-dose betaine (betaine hydrochloride 10g/day), vitamin B12, methionine, and FA [24]. The patient experienced complete recovery of vision with impressive improvement in cognition and gait. In fact, in pediatric cases with severe MTHFR deficiency, betaine has demonstrated efficacy in averting mortality and enhancing psychomotor development [24,25].

A few other studies have explored the possible treatment options for HHCY. In a meta-analysis, it was suggested that daily folic acid supplementation ranging from 0.5 to 5 mg contributes to a reduction in HHCY by approximately 25%, while in a randomised controlled trial the reduction was found to be 21% [26]. While in one other randomised controlled trial the reduction in HHCY with 200 μg each of folate, 5-Me-THF and folic acid supplementation was found to range between 19-21% [27]. The addition of vitamin B12 (at an extra 0.5%) was found to further decrease the concentration of HCY by an additional 7%. Another study reported that supplementation with vitamin B12 and folate led to reductions in HCY levels by 7% and 23%, respectively. Specifically, folate, vitamin B6 and vitamin B12 were observed to decrease HCY levels by 43%, 12%, and 5%, respectively [4,17]. Nevertheless, in their experimental study, Golja et al. assessed the influence of folate deficiency and augmentation on the cell growth and proliferation of human lymphoblastoid cells [15]. Their objective was to assess the efficacy of FA or 5-Me-THF supplementation in enhancing the intracellular concentration of biologically active folate across genetically diverse cells. Two MTHFR activity groups were delineated, aligning with previous studies indicating that compound heterozygosity for 677CT/1298AC yields a comparable clinical significance to C677T homozygosity. The findings indicated that individuals with reduced MTHFR activity derived benefits from 5-Me-THF supplementation. This outcome was anticipated, as 5-Me-THF operates independently of MTHFR's ability to metabolize folate effectively. Nonetheless, a significant amount of FA might be able to circumvent conversion issues arising from MTHFR polymorphisms, therefore, the evidence supporting the ability of 5-Me-THF to overcome folate insufficiency resulting from MTHFR deficiency is of utmost significance, given that a significant portion of the population cannot effectively metabolize folate due to the MTHFR enzyme defects they carry. In these cases, supplementing with 5-Me-THF demonstrated greater efficiency compared to FA supplementation, as it can directly integrate into the folate cycle without requiring enzymatic modification, potentially overcoming metabolic defects. Furthermore, it aids in mitigating the concern that high-dose folate supplementation poses potential risks, with a notable risk being the masking of vitamin B12 deficiency, which could lead to severe neurological effects. Elevated folate levels might also be linked to insulin insensitivity in offspring, an increased likelihood of infants being small for gestational age, and an elevated risk of certain cancers, depression, and cognitive impairment. The conclusion drawn was that FA undergoes an intricate metabolism, and its impacts are contingent on the levels of the biologically active form, 5-Me-THF. The enzyme 5,10-MTHFR, crucial in the folate pathway for synthesizing this biologically active form, displays notable polymorphism, with around 90% of the European population carrying polymorphic variants of MTHFR [15].

Several other clinical studies have highlighted a notable correlation between MTHFR polymorphisms and a range of conditions, including severe congenital birth defects like neural tube defects, congenital heart defects and orofacial clefts. Additionally, cardiovascular diseases, neuronal development disorders, cancers, and psychiatric conditions have been identified as having a significant association with MTHFR polymorphisms. The MTHFR gene exhibits significant polymorphism, with the most extensively studied variants that reduce MTHFR activity being 677C>T (rs1801133) and 1298A>C (rs1801131) [15].

Limitations

The identified studies were all conducted with variable sample sizes and had some heterogeneity in terms of their outcomes. This study was also limited in its course of duration including only papers from the last 10 years and its English language preference. The impact of gene polymorphism needs to be explored more with better sample size and more large-scale studies across all populations. This study also lacks the data for the recurrence rate of IS in those diagnosed with MTHFR gene C677T mutation. More studies need to be conducted to create awareness about the pathophysiology of stroke with the intention of not only diagnosing and treating but also preventing, a simple yet challenging etiology of the disease and its dreaded outcome in young patients. 

## Conclusions

This systematic review aimed to investigate the association between MTHFR gene C677T polymorphism, leading to HHCY, and the occurrence of IS. Additionally, the review sought to shed light on potential strategies for the early diagnosis of arterial dissection in young adults, which is sometimes a consequence of HHCY. The findings from the reviewed articles indicated that the initial symptoms of dissections often resemble those of migraine or cervicogenic headache, potentially leading to misdiagnosis in young individuals. Therefore, maintaining a high clinical suspicion is crucial when individuals present with first-time symptoms, suggestive of migraine or cervicogenic headache. Furthermore, our analysis led us to the conclusion that supplementation with vitamin B12, B6, and folate can significantly benefit individuals with gene polymorphisms, aiding in the management of HHCY. Notably, the methyl donor betaine has demonstrated remarkable effectiveness, particularly in individuals with severe MTHFR deficiency. However, it is crucial to note that further extensive studies and trials are required for widespread acceptance of this approach.
