# Correction: Exploring the Nexus: A Systematic Review on the Interplay of the Methylenetetrahydrofolate Reductase (MTHFR) Gene C677T Genotype, Hyperhomocysteinemia, and Spontaneous Cervical/Vertebral Artery Dissection in Young Adults

**DOI:** 10.7759/cureus.c276

**Published:** 2025-08-26

**Authors:** Sajida Moti Wala, Esraa M AlEdani, Essa A Samuel, Khoula Ahmad, Naelijwa J Manongi, Ramkumar Rajapandian, Safeera Khan

**Affiliations:** 1 Internal Medicine, California Institute of Behavioral Neurosciences & Psychology, Fairfield, USA; 2 Dermatology, California Institute of Behavioral Neurosciences & Psychology, Fairfield, USA; 3 Physical Medicine and Rehabilitation, California Institute of Behavioral Neurosciences & Psychology, Fairfield, USA; 4 internal Medicine, California Institute of Behavioral Neurosciences & Psychology, Fairfield, USA; 5 Family Medicine, California Institute of Behavioral Neurosciences & Psychology, Fairfield, USA; 6 Trauma and Orthopaedics, California Institute of Behavioral Neurosciences & Psychology, Fairfield, USA

This article has been corrected to fix the following errors:

- Abstract: "This systematic review encompasses a thorough analysis of 11 papers, including **four observational studies, **three case reports**, **three narrative reviews, and one experimental study, involving 4,840 patients aged 18-**45** years." has been corrected to "This systematic review encompasses a thorough analysis of 11 papers, including **two case-control studies, one retrospective observational study, one cohort study**, three case reports, three narrative reviews, and one experimental study, involving a total of 4,840 patients aged 18–**64** years."
- Results: "We shortlisted a total of **108** articles..." has been corrected to "We shortlisted a total of **107** articles..."
- PRISMA diagram:
  - "Reports sought for retrieval (n=**124**)" has been corrected to "Reports sought for retrieval (n=**123**)"
  - "Reports assessed for eligibility (n=**108**)" has been corrected to "Reports assessed for eligibility (n=**107**)"
  - "Reports excluded: Other reasons (n=**35**)" has been corrected to "Reports excluded: Other reasons (n=**33**)"

